# Supplementary material for: Inhibition of the succinyl dehydrogenase complex in acute myeloid leukemia leads to a lactate-fuelled respiratory metabolic vulnerability
Source: Nat Commun. 2022 Apr 19;13:2013. doi: 10.1038/s41467-022-29639-0 (PMC9018882; doi:10.1038/s41467-022-29639-0)
Supplement: Supplementary file 1 — Supplementary Information [file 41467_2022_29639_MOESM1_ESM.pdf]

Supplementary material

Inhibition of the succinyl dehydrogenase complex in Acute Myeloid Leukemia leads to a lactate-fuelled respiratory metabolic vulnerability

Ayşegül Erdem[1,2], Silvia Marin[2,3,4], Diego A. Pereira-Martins[1,5],

Marjan Geugien[1], Alan Cunningham[1], Maurien G. Pruis[1], Isabel Weinhäuser[1,5],

Albert Gerding[6,7], Barbara M. Bakker[7], Albertus T.J. Wierenga[1,6], Eduardo M. Rego[5],

Gerwin Huls[1], Marta Cascante[2,3,4,8] and Jan Jacob Schuringa[1,8,9]

[1]Department of Experimental Hematology, University Medical Center Groningen, University of Groningen, Hanzeplein 1, 9700 RB, Groningen, The Netherlands.

[2]Department of Biochemistry and Molecular Biomedicine, Faculty of Biology, University of Barcelona, Avda. Diagonal 643, Barcelona 08028, Spain.

[3]CIBER of Hepatic and Digestive Diseases (CIBEREHD), Institute of Health Carlos III, 28029 Madrid, Spain. [4]Institute of Biomedicine of University of Barcelona, 08028 Barcelona, Spain. [5]Hematology Division, LIM31, Faculdade de Medicina, University of São Paulo, São Paulo, SP, Brazil. [6]Department of Laboratory Medicine, University Medical Center Groningen, University of Groningen, Hanzeplein 1, 9700 RB, Groningen, The Netherlands. [7]Laboratory of Pediatrics, Section Systems Medicine of Metabolism and Signaling, University Medical Center Groningen, University of Groningen, Groningen, The Netherlands. [8]Senior authors. [9]Lead contact.

Supplementary Figures

Supplementary Figure 1

a

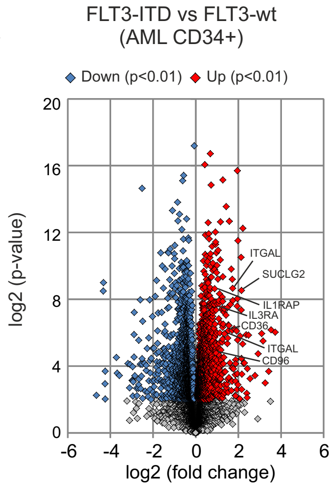

b

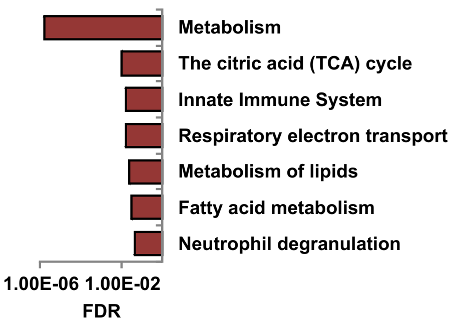

c

NADH regeneration/oxidation		Electron Transport Chain
ACADVL	HSD17B12	COX5B
AGPS	HSD17B4	COX6B1
ALDH1A1	IDH1	ETFDH
ALOX5	ME2	NDUFA12
CPOX	PGD	NDUFA7
CPT1A	PPOX	NDUFA9
CYP27A1	PTGES2	NDUFB7
FDX1L	RBKS	NDUFS1
FH	RETSAT	NDUFS2
GLUD1	SUCLG1/2	NDUFV1
HADHA	SUMF1	NDUFV2
HADHB	TBXAS1	
HIBCH		

d

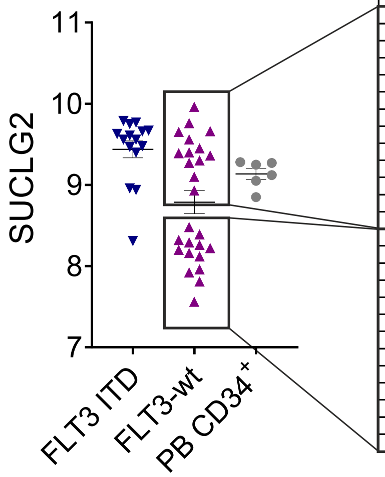

Sample Nr	SUCLG2	FLT3	NPM	karyotype	mutations
28	9.96	wt	cyt	NK	IDH1, PTPN11, NPM
35	9.76	wt	cyt	NK	
31	9.66	wt	cyt	NK	IDH2, NPMcyt
17	9.65	wt	wt	45 xx, 3p+, -7, 8p-, 46xx	
23	9.56	wt	wt	NK	WT1
32	9.45	wt	wt	inv16	
19	9.40	wt	wt	NK	IDH2, RUNX1, SFSR2
18	9.39	wt	wt	inv (16)(p13q22)/t(16;16)(p13;q22) [CBFß/MYH11].	
25	9.36	wt	wt	inv 16	KRAS, IDH1 R132H (2%)
20	9.30	wt	wt	t(9;22)/inv 16	MLL2, IDH1 R132H (4%)
29	9.27	wt	wt	NK	
37	9.10	wt	wt	MLL-AF4	SET2D, IDH1 R132H (2%)
34	8.93	wt	wt	NK	ASXL1, NRAS, RUNX1
22	8.48	wt	wt	NK	
30	8.39	wt	wt	NK, IDH2 R140Q (original seq)	ASXL1, IDH2, SRSF2
40	8.32	wt	wt	NK, CEBPa mut	
33	8.29	wt	wt	NK; JAK2 mut	
36	8.26	wt	wt	NK, CEBPa mut	
41	8.22	wt	wt	NK	
26	8.20	wt	n.a.	atyp CML	
27	8.16	wt	wt	complex	
24	8.12	wt	wt	NK	CEBPa, EP300, SRSF2, TET2
21	7.96	wt	wt	NK	NRAS (0.25), WT1 (0.2), CEBPA (0.46), IDH1 R132H (1%)
39	7.92	wt	wt	t(2;3)	
38	7.81	wt	wt	IDH2 R140Q, +11	IDH2, SRFS2, IDH1 R132H (3%)
16	7.56	wt	wt	-9q	

e

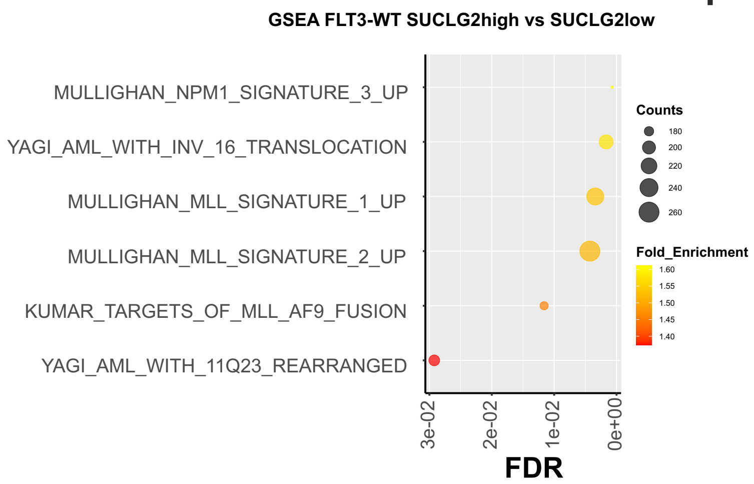

f

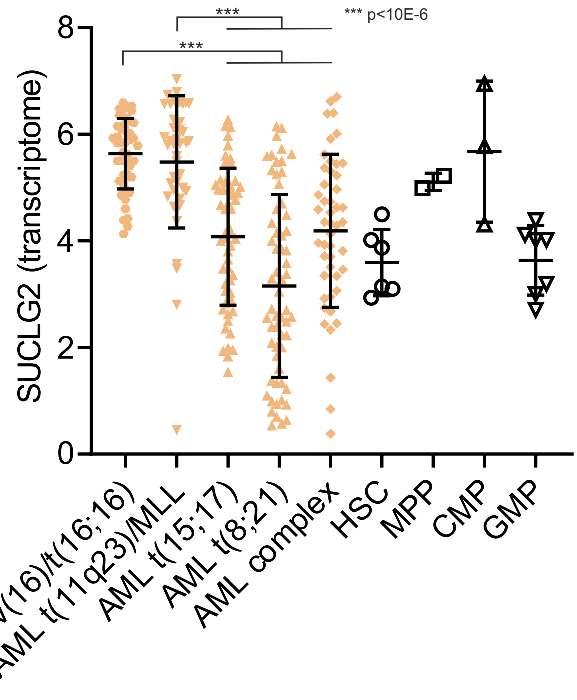

Supplementary Fig.1. *FLT3*-ITD mutant AMLs express more OXPHOS-driven metabolic profiles compared to *FLT3*-wt AMLs and detailed analyses on *FLT3*-WT/SUCLG2^{high} cells. (a) Volcano chart shows comparison of label-free quantitative proteome analyses of the CD34^{+} *FLT3*-wt (n = 26) and *FLT3*-ITD^{+} (n = 15) AML patients. Significantly (p<0.01) up-regulated proteins in *FLT3*-ITD^{+} AML primary cells are shown in red and down-regulated proteins are shown in blue; x-axis indicate log2 fold changes and y-axis indicate p-value in –log2. Adjusted p-values were calculated using the online Galaxy Limma-Vroom tool using Benjamini and Hochberg multiple testing correction. (b) Bar chart shows list of highly enriched metabolism-related gene ontology (GO) terms named and ranked in the y-axis and x-axis shows FDR (false discovery rate) p-values. (c) Individual list of the proteins highly enriched in following GO terms; NADH regeneration/oxidation and electron transport chain of *FLT3*-ITD^{+} patients. (d) SUCLG2 proteome data was ranked from high to low, and the *FLT3*-WT/SUCLG2^{high} patient population contained all the NPMcyt and inv16 cases (*FLT3*-ITD^{+} n = 15, *FLT3*-wt n = 26, PBSC n = 6). (e) Pearson coefficients were calculated using the quantitative proteome comparing the *FLT3*-WT/SUCLG2^{high} group with the *FLT3*-WT/SUCLG2^{low} group, and the ranked list was used for GSEA. (f) SUCLG2 expression in the MILE cohort (GSE13159) compared to expression in normal HSCPs (GSE42519) (AML t(15;17): n = 54; AML inv(16)/t(16;16), n = 47; AML t(8;21): n = 60; AML t(11q23)/MLL: n = 43; AML complex: n = 48; HSC: n = 6, MPP: n = 2; CMP: n = 3, GMP: n = 7) (Student's t-test (two-sided)).

Supplementary Figure 2

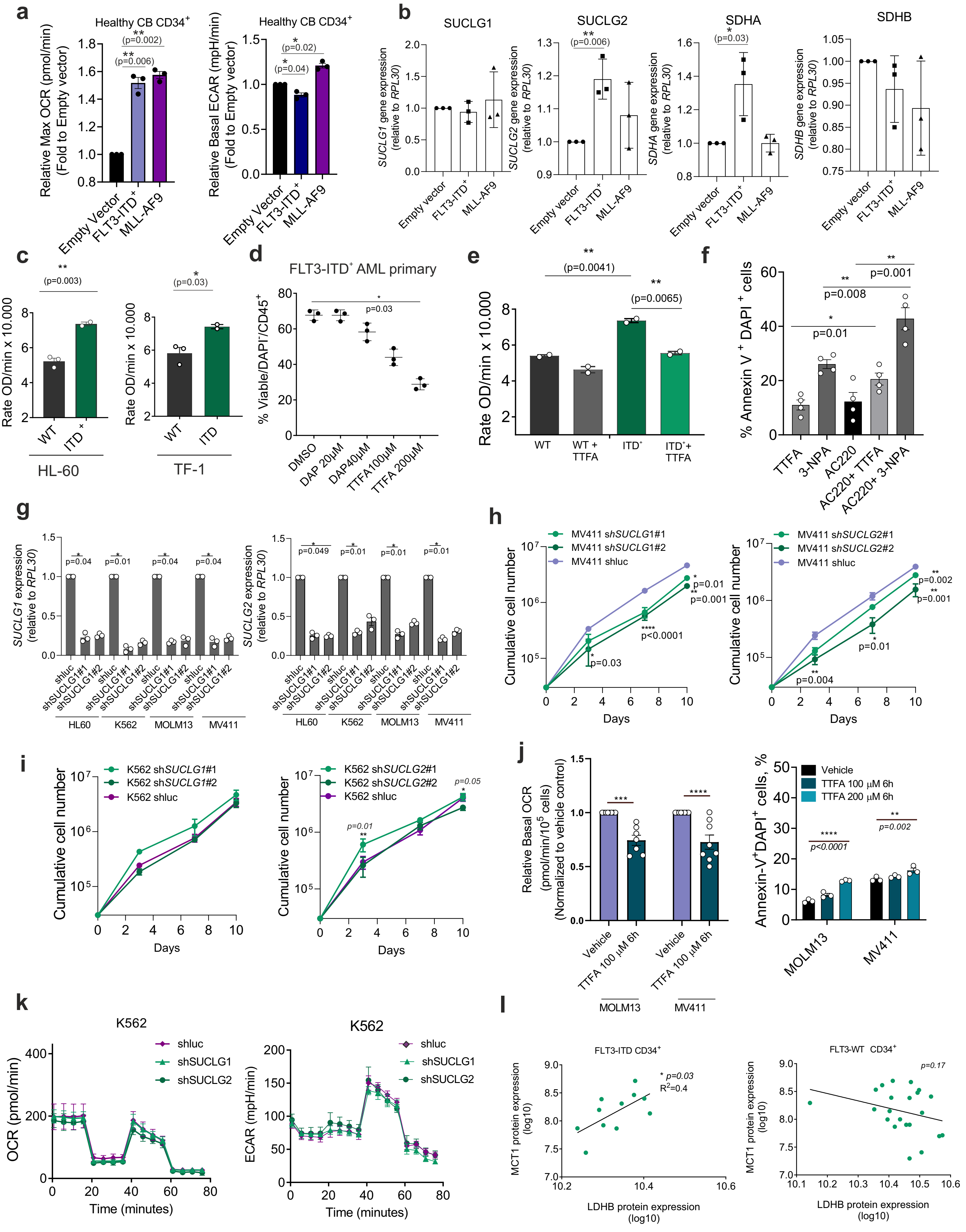

Supplementary Fig. 2. *FLT3*-ITD$^+$ association with Complex II activity, OXPHOS and lactate influx.

(a) OCR and ECAR measurements in CB CD34$^+$ cells transduced with *FLT3*-ITD or *MLL-AF9* (sorted) (n = 3 measured in 4 technical replicates (mean +/- SEM)). (b) Q-RT-PCR data in transduced CB CD34$^+$ cells expressing *FLT3*-ITD or MLL-AF9 (sorted) (n = 3 measured in 3 technical replicates (mean +/- SEM)). (c) ETC Complex II enzymatic activity levels measured in HL60 GFP$^+$ sorted cells (left panel) and in TF1 GFP$^+$ sorted cells (right panel) after being transduced lentivirally with empty vector with GFP (WT) and ITD$^+$-GFP overexpression constructs n = 2 measured in two technical replicates (mean +/- SEM). (d) % DAPI$^-$ viable CD34$^+$ AML patient cells (n = 1 *FLT3*-ITD) treated with ETC Complex II inhibitor TTFA (100, 200 μM) or DAP (20, 40 μM) for 48 hours. Data generated by flow cytometry (n=3, each dot shows a technical replicate (mean +/- SEM)). (e) ETC Complex II activity levels measured using spectrophotometry in HL60 GFP$^+$ sorted cells cells after lenti-viral transduction with empty vector with GFP (WT) and ITD$^+$-GFP overexpression constructs and then treated with/without 200 μM TTFA for 24 hrs (n = 2 measured in two technical replicates). (f) (%) Annexin V$^+$DAPI$^+$ MOLM13 cells upon ETC Complex II inhibitors (TTFA 100 μM and 3-nitropropionic acid 2 mM, indicated as 3-NPA) with/without 5 nM AC220 for 24 hours. Data generated by flow cytometry. Each bar represents mean +/- SEM of three biological replicates (each measured in three technical replicates). (g) SUCLG1/2 mRNA expression in AML cell lines with shluc, sh*SUCLG1#1*/2 or sh*SUCLG2*#1/2 (n = 3 measured in 3 technical replicates (mean +/- SEM)). (h-i) Cumulative cell growth of MV411 (h) and K562 (i) cells transduced with shluc, sh*SUCLG1*#1, sh*SUCGL1*#2, sh*SUCLG2*#1, sh*SUCLG2*#2 (n = 3

measured in 3 technical replicates (mean +/- SEM)). (j) OCR (left panel) and apoptosis (right panel) levels after 6 hours TTFA treatment in MOLM13 and MV411 cells (n = 3 measured in 4 technical replicates (mean +/- SEM)). (k) OCR and ECAR levels in shluc, sh*SUCLG1*#1, sh*SUCGL1*#2 , sh*SUCLG2*#1, sh*SUCLG2*#2 K562 cells. Equal number of viable cells were screened (n = 3 measured in 4 technical replicates (mean +/- SEM)). (l) Protein expression (log 10) of MCT1 compared to LDHB in *FLT3*-ITD^{+} (left panel) and *FLT3*-wt (right panel) primary AML patient samples.

(a, b, c, e, f) Student's t-test (two-sided), (d, g) one-way ANOVA or (h,i,j,k) two-way ANOVA for multiple comparisons or (l) linear regression analysis.

Supplementary Figure 3

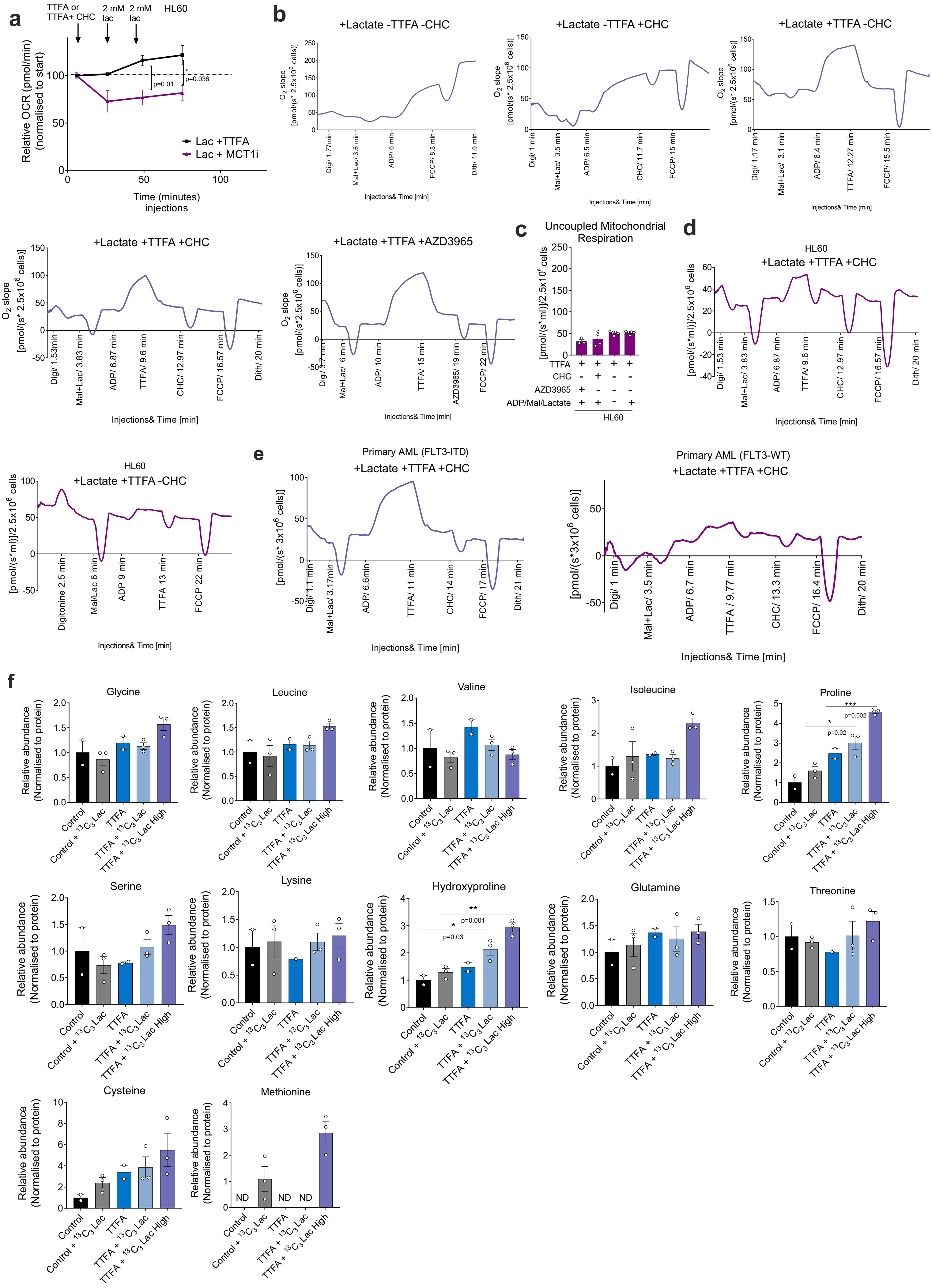

Supplementary Fig. 3. Internal metabolite abundances determined by GC-MS analysis in MOLM13 cells with labelled [$^{13}C_3$] lactate.

(a) Real time OCR in HL60 cells. Either 600 μM TTFA or 600 μM TTFA with 600 μM CHC were injected at the start of the measurement and then 2mM sodium lactate was injected every 25 minutes serially. Data normalized to the initial OCR at 100. Data generated from four independent biological replicates and plotted as the mean of each biological replicate (mean +/- SEM), in quadruplicate repeats. (b) Real-time high-resolution respirometry analysis in 2.5 x 10^6 MOLM13 cells (37°C) in the presence or absence of sequentially injected (every 3-5 minutes after respiration is stable); Digitonin (Digi) (2.5 μg/ml), Malate (2 mM) + Lactate (8 mM), ADP (1mM), TTFA (175 μM), CHC (400 μM), (or AZD3965 (150 nM), FCCP (1.5 μM) and Dithionite (0.05%). O_2 consumption levels were calibrated according to the basal levels of the chambers. Data shows raw measurements. (c) Real-time high-resolution respirometry measurement in HL60 cells in the presence/absence of sequentially injected (every 3-5 minutes after respiration is stable) Digitonin (2.5 μg/ml), Malate (2 mM) with/without Lactate (8 mM), ADP (1mM), TTFA (175 μM), CHC (400 μM), (or AZD3965 (100 nM)), FCCP (1.5 μM) (2.5 x 10^6 cells/ml). After digitonin, Malate and ADP are used at the beginning and FFCP is used at the end of all measurements as major respiration substrates. Bar graphs demonstrate end-point respiration levels after an ETC-uncoupler FCCP was injected according to each condition combination indicated below the x-axis (n = 3 biological replicates, mean +/- SEM). (d) As in (b) but now for HL60 cells. (e) Real-time high-resolution respirometry analysis performed in 3 x 10^6 AML patient cells in the presence of sequentially injected (every 3-5 minutes after respiration was stable); Digitonin (2.5 μg/ml), Malate (2 mM) + Lactate (8 mM), ADP (1mM), TTFA (175 μM), CHC (400 μM), FCCP (1.5 μM) and Dithionite

(0.05%). Data shows raw measurements of two independent biological replicates. (f) Internal relative abundance of TCA cycle associated metabolites in MOLM13 cells were grown with/without 200 μM TTFA and with/without 8 mM and 20 mM labelled [$^{13}C_3$] lactate **(**6h). Data was normalized to norvaline as internal recovery standard and to protein amount. (mean+/-SEM of three independent replicates).

(a,c,f) Student's t-test (two-sided).

Supplementary Figure 4

a

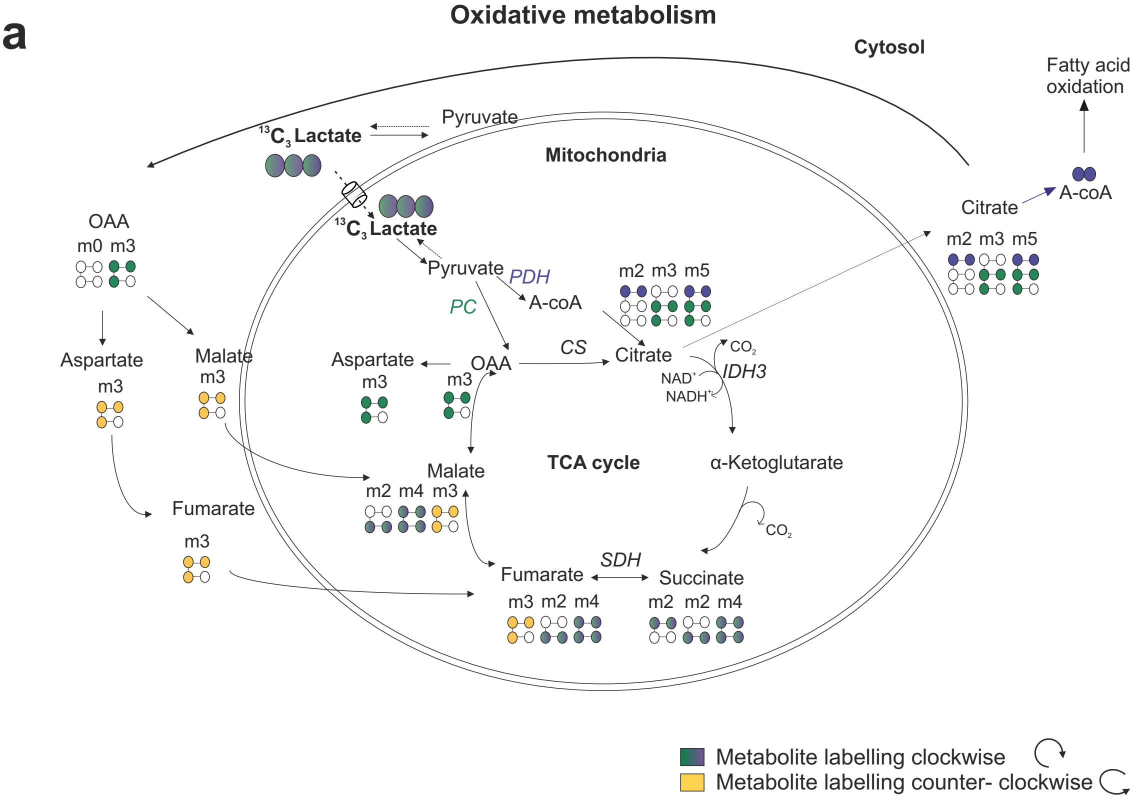

b

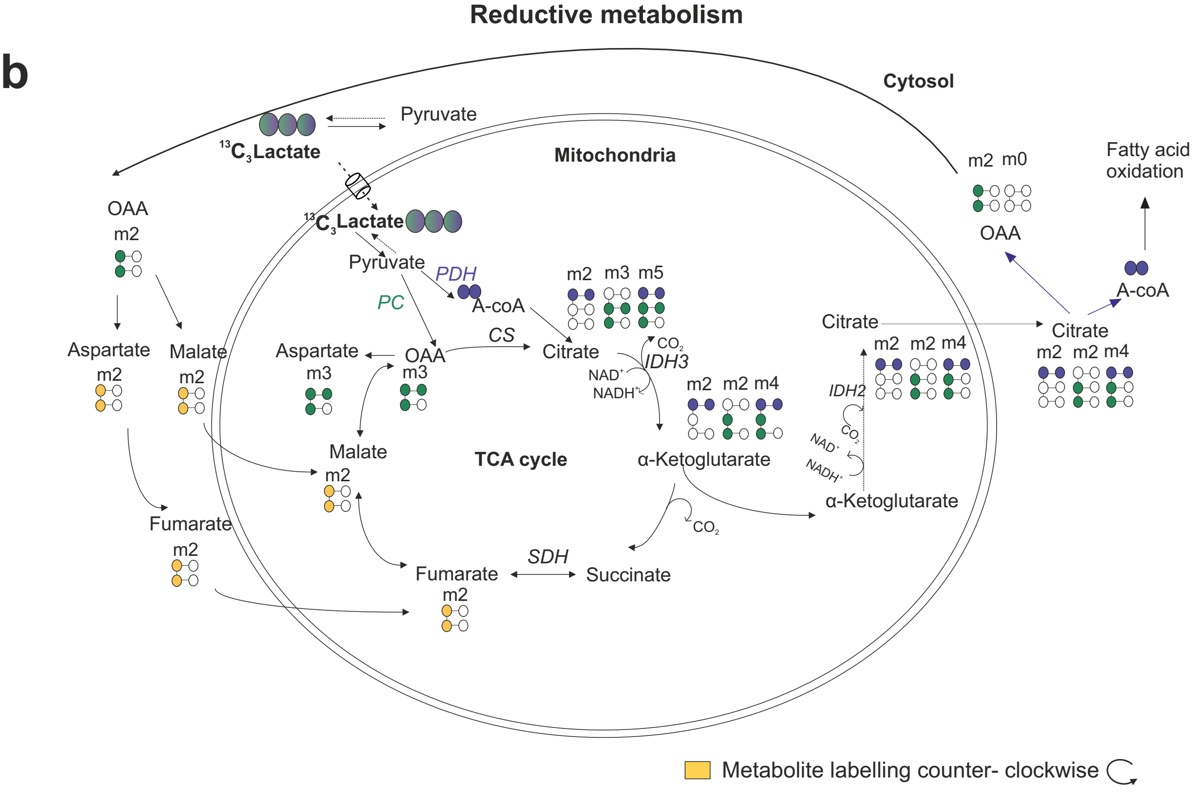

Supplementary Fig. 4. Mass isotopologue flux analysis from [$^{13}C_3$] lactate.

Schemes demonstrate labelling pattern of the TCA cycle intermediates in MOLM13 cells cultured with [U-$^{13}C_3$] lactate for 6 hours. Lactate contributes to fuelling TCA cycle both in control and TTFA treated cells and is metabolized through PDH (in blue) and PC (in green) to generate citrate. Upper panel shows metabolite labelling in oxidative metabolism and lower panel shows metabolite labelling in cytosolic reductive metabolism. Green&blue (starts with [U-$^{13}C_3$] lactate) and yellow circles are coloured depending on if the label obtained is the result of being the metabolites metabolized by TCA-cycle enzymes clockwise or counter-clockwise, respectively. (a) Oxidative metabolism reactions involve IDH3 in the TCA cycle, transiting citrate through α-ketoglutarate (α-KG). The citrate generated via PDH and PC (m+3 and m+5 labelled) can be exported out of the mitochondria resulting in m+3 and m+5 labelling in the cytosol and generates m3 labelling in the downstream metabolites. (b) Reductive metabolism reactions are combinations of oxidative metabolism and cytosolic citrate metabolism. The citrate (m+2 and m+4 labelled) generated from α-ketoglutarate (α-KG) via the IDH2 enzyme inside the mitochondria is exported to the cytosol. Cytosolic products of citrate are m+2 labelled fumarate, aspartate and malate (counter-clockwise in yellow). The significantly increased labelling pattern observed in m+2 malate and m+2 aspartate (yellow) upon SDH inhibition in Fig. 6, are derived via the cytosolic reductive carboxylation route from cytosolic citrate.

Supplementary Figure 5

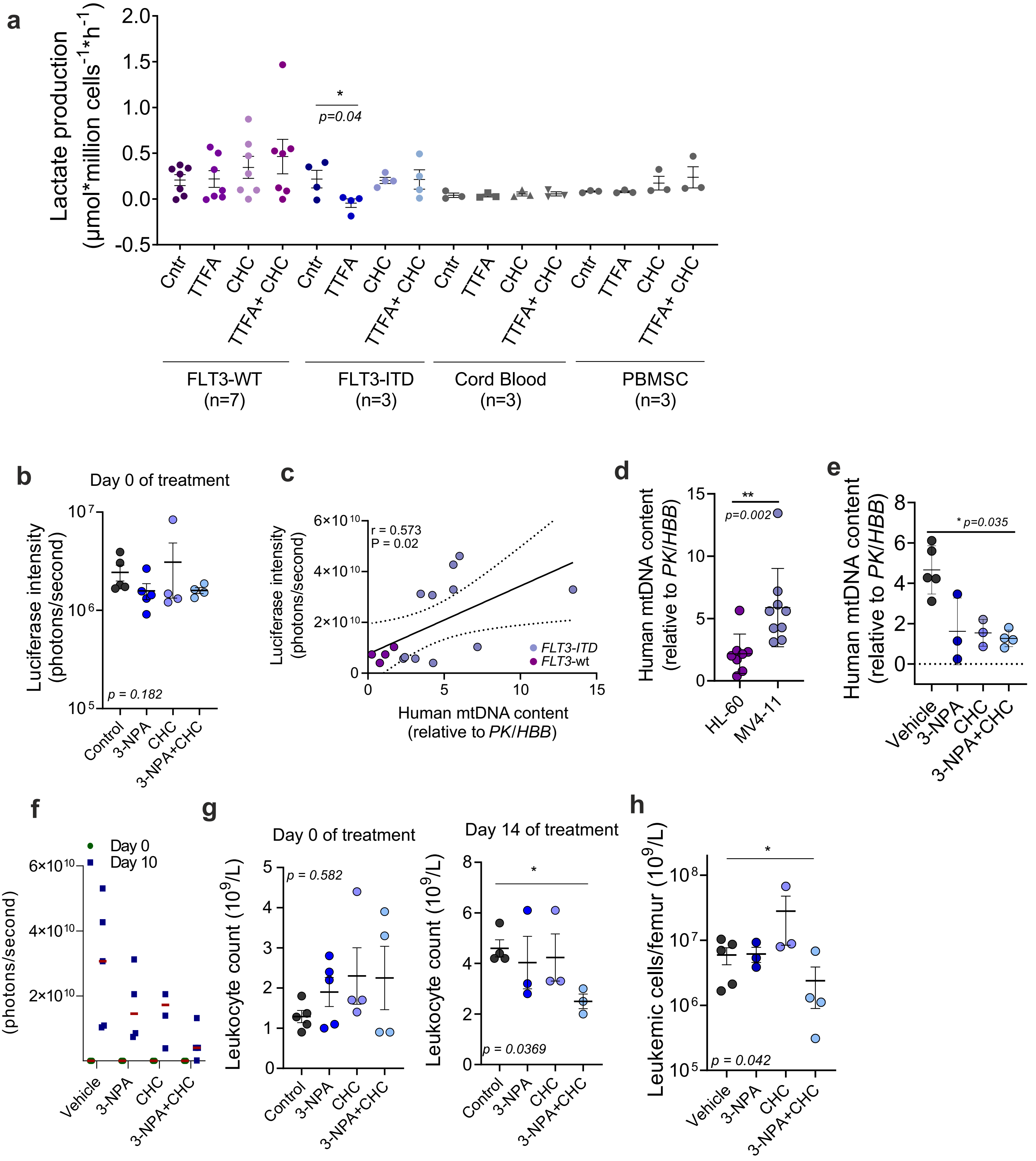

Supplementary Fig. 5. Extended data of lactate flux rate levels and in vivo assays.

(a) Flux of lactate secreted to the media, corrected by cell and incubation time, measured in $CD34^+$ sorted AML primary (n = 7 *FLT3*-wt, n = 3 *FLT3*-ITD), Healthy Cord Blood (CB) (n = 3), Healthy Peripheral Blood Mobilized Stem Cells (PBMSC) (n=3) in the presence of ETC Complex II inhibitor (TTFA 100 μM with/without 800 μM CHC for 48 hrs. Each data point represents mean +/- SEM independent biological replicates (each dot) measured in four technical replicates. (b) Luciferase intensity in mice transplanted with MV411 cells which were randomly assigned to four groups at the start of the treatment. Each data point represents mean +/- SEM (n = 5). (c-e) Correlation (c) and quantification (d-e, (mean +/- SEM)) between human mtDNA content from the peripheral blood after confirmation of the engraftment by luciferase intensity measurement in both HL60 (dark purple, n = 9) and MV411 (blue, n = 9) transplanted mice. In e, samples were compared from vehicle versus compound-treated mice (n = 5). (f) Mean luciferase intensity was also less pronounced at day 10 after combination treatment compared to vehicle group. (g-h) The combination of 3-NPA and CHC treatment significantly reduced blood leukocyte counts in mice transplanted with MV411 (g) and leukemic cell counts per femur (h). (n = 5 in each group (mean +/- SEM)).

(a, d) Student's t-test (two-sided), (b,e,f,g,h) two-way ANOVA for multiple comparisons or (c) linear regression analysis.
