# Supplementary material for: Inhibition of the succinyl dehydrogenase complex in acute myeloid leukemia leads to a lactate-fuelled respiratory metabolic vulnerability
Source: Nat Commun. 2022 Apr 19;13:2013. doi: 10.1038/s41467-022-29639-0 (PMC9018882; doi:10.1038/s41467-022-29639-0)
Supplement: Supplementary file 3 — Description of Additional Supplementary Files [file 41467_2022_29639_MOESM3_ESM.pdf]

Description of Additional Supplementary Files

Supplementary Data 1: Patient Information and OS

Supplementary Data 2: LFQ Proteome data

Supplementary Data 3: Mass isotopologue distributions
